# Staircase-Enhanced Magneto-Electric Dipole Antenna for Wideband CP 5G Applications with High-Gain Arrays

**DOI:** 10.3390/s25247620

**Published:** 2025-12-16

**Authors:** Hend Malhat, Amer Zakaria, Nasser Qaddoumi

**Affiliations:** Department of Electrical Engineering, American University of Sharjah, Sharjah P.O. Box 26666, United Arab Emirates; hmalhat@aus.edu (H.M.); nqaddoumi@aus.edu (N.Q.)

**Keywords:** magneto-electric dipole, circular polarization, MIMO, sequential array, fifth-generation, 5G

## Abstract

This paper presents a compact magneto-electric dipole (MED) antenna optimized for wideband circularly polarized (CP) radiation for 5G applications. It incorporates a staircase-shaped electric dipole with trimmed corners to excite orthogonal modes for enhanced CP performance. The proposed single-layer MED antenna achieves a 20.6% wide-impedance bandwidth ($|S_{11}|\;<-10$ dB, 22.97–28.12 GHz) and 21.9% CP bandwidth (AR<3 dB, 22.23–27.83 GHz) with a compact footprint of $15\times 15\times 1.6\;{\mathrm{mm}}^{3}$. There is a symmetrical radiation pattern with a co-to-cross polarization ratio >23 dB and a stable gain of 8.8 dBi. An equivalent circuit model is optimized via particle swarm optimization (PSO). The optimized MED antenna is utilized to investigate various CP-MIMO configurations and wideband sequential arrays. Next, a 1×2 CP-MIMO antenna system is developed, employing polarization diversity in parallel and mirror configurations. Isolation is improved by etching a ground slot between the MED elements, yielding isolation levels of below −20 dB and −23 dB, respectively. Further, a 2×2 CP-MIMO configuration is designed and evaluated. This arrangement demonstrates an envelope correlation coefficient (ECC) of $1\times 10^{-3}$ and a diversity gain of approximately 10 dB across the operating bandwidth. Finally, a sequential array is designed that applies a $90^{\circ }$ sequential rotation and phase excitation to MED elements for high-gain CP 5G communications. Here, various array sizes are evaluated, with an 8×8 MED array providing CP radiation (AR≤1 dB) from 20 to 30 GHz with enhanced impedance and axial ratio bandwidths and stable gain with a peak value of 27.47 dBi.

## 1. Introduction

Fifth-generation (5G) wireless communication systems are designed to deliver high-speed data transmission, ultra-low latency, and massive connectivity [1]. 5G communication uses various frequency bands, including the sub-6 GHz and millimeter-wave (mmWave) bands, which enable significantly higher data rates than traditional cellular frequencies. This capability supports various applications, including enhanced mobile broadband, healthcare, Internet of Things (IoT), autonomous vehicles, and high-definition video streaming for augmented and virtual reality interactions [2]. However, challenges in 5G mmWave communications include significantly higher free-space path loss and being more susceptible to attenuation from obstacles and atmospheric conditions [1].

Antennas are crucial in 5G systems as they are designed to support increased bandwidth requirements, facilitating rapid data exchange and enabling the simultaneous connection of multiple devices. However, these antennas would work within shorter communication ranges and be more susceptible to obstacles, such as buildings and trees. To address the challenges of 5G networks, advanced techniques such as beamforming with high-gain arrays and MIMO (Multiple-Input Multiple-Output) are employed [3]. Beamforming using antenna arrays directs the signal in specific directions, thereby enhancing coverage and signal strength. High-gain antennas are employed to focus the radiated energy into a narrower beam, thereby increasing the signal strength over longer distances and improving the signal-to-noise ratio. Antenna arrays are designed to achieve such high-gain radiation by optimizing the feeding network, which ensures precise phase and amplitude distribution across their elements. MIMO technology represents a significant advancement in data transmission and reception for modern wireless communication standards like LTE, Wi-Fi, and 5G. By utilizing multiple antennas at both the transmitter and receiver ends, MIMO systems exploit spatial diversity and multipath propagation [4]. This leads to substantial improvements in data throughput, reliability, and spectral efficiency compared to traditional single-input, single-output (SISO) systems [5]. These enhancements enable higher data rates, improved coverage, and better user experiences in dense urban environments and challenging propagation conditions.

In this paper, a low-profile wideband circularly polarized (CP) magneto-electric dipole (MED) antenna is proposed and implemented for a 1×2 and 2×2 MIMO systems, and an 8×8 sequential array for mmWave 5G communications. The shape and dimensions of the CP-MED antenna are optimized to achieve a wideband CP radiation over the operating bandwidth. Next, the arrangements of two and four individual antenna elements in the MIMO system are investigated to achieve the best performance. Finally, high-gain radiation is achieved by designing a 2×2, 4×4, and 8×8 MED sequential array for wideband CP communications, resulting in enhanced performance.

The following sections give a brief literature review of MED antennas and configurations in MIMO systems. This is followed by Section 2, where the design methodology of a novel MED antenna is shown along with the simulation, equivalent circuit, and implementation results. In Section 3, a numerical investigation is carried out to design an MIMO antenna system. This is followed by a MIMO diversity performance analysis in Section 4. Next, the radiation characteristics of a high-gain sequential MED array are investigated in Section 5. The paper is concluded in Section 6.

### 1.1. Magneto-Dipole Antennas

A MED antenna combines the properties of both electric dipole and magnetic dipole antennas to achieve broad bandwidth, stable radiation patterns with low cross-polarization levels, and improved efficiency [6]. It consists of an electric dipole component, a magnetic dipole component, and a proximity-coupled feeder. MED antennas are ideal for wireless communication systems, radar, and satellite systems applications. In [7], a low-profile, dual-polarization MED antenna with dual orthogonal polarization and 36.8% bandwidth was proposed and investigated for 5G applications. CP antennas are preferred in wireless communication systems because they reduce polarization mismatch losses, enhance signal reliability, and improve quality [8]. They offer consistent and reliable performance in changing environments. Further, in [9], a compact CP-MED antenna with a 24.6% impedance matching bandwidth, 18.1% CP bandwidth, and a peak gain of 8 dBi. Another MED antenna based on substrate-integrated waveguide (SIW) technology featuring an aperture-coupled feeder with a CP bandwidth of 12.8% was also developed in [10].

### 1.2. MIMO Configurations

MIMO-CP antennas are preferred in communication systems as they are particularly effective in improving signal quality and reliability, especially in scenarios where the orientation of transmitting and receiving devices varies. Additionally, MIMO-CP systems achieve higher data rates and better link reliability through spatial multiplexing and diversity techniques [11]. Despite the benefits of MIMO systems, the performance of closely spaced antenna elements in such systems can be influenced by the elements’ proximity and electromagnetic interactions [12]. Mutual coupling affects the system’s diversity gain, overall capacity, and the effective number of independent channels available for data transmission. Various techniques are employed to mitigate the mutual coupling effect in MIMO systems.

An example of such techniques is the incorporation of decoupling structures between elements [12]. These decoupling structures include defected ground structures (DGS), parasitic elements, metamaterials, or orthogonal polarizations. In [13], a planar MIMO-CP patch antenna with high isolation ($|S_{21}|<-20$ dB) using F-shaped DGS has been proposed. Furthermore, in [14], four parasitic elements were inserted between two MIMO-CP diagonal slotted patches to improve isolation ($|S_{21}|<-22$ dB) and to achieve a gain of 7.5 dBi. Another method to reduce mutual coupling effects is the polarization diversity technique, which uses different polarization orientations for the MIMO elements. This includes using elements with orthogonal polarizations, which involves orienting antennas in different polarization planes, reducing the coupling effect as the antennas receive and transmit signals in different polarizations. For instance, in [15], a two-port microstrip MIMO-CP array designed for WLAN applications achieved an impedance bandwidth ranging from 5.23 to 6.42 GHz, an axial ratio of less than 3 dB from 5.37 to 5.72 GHz, and high isolation of 37 dB.

Furthermore, MIMO systems utilizing decoupling structures and polarization diversity have been reported. For example, in [16], a planar dual-port MIMO antenna array with left-hand circular polarization (LHCP) and right-hand circular polarization (RHCP) features has been reported for 5G sub-6 GHz applications. Here, an optimized Z-shaped slot-loaded DGS was designed to achieve high isolation and enhanced MIMO performance parameters, including an envelope correlation coefficient (ECC) less than 0.005 and a diversity gain (DG) of approximately 9.99 dB, with a mean effective gain (MEG) less than 3 dB. Another example is presented in [17], where a polarization-reconfigurable MIMO antenna array was designed using a diagonal slotted cylindrical patch integrated with PIN diodes and a T-shaped power divider. Here, a sinusoidal-like DGS etched in the ground provided port isolation of 30 dB over the operating band. The MIMO array covered the 25.2–29.4 GHz band and achieved a peak gain of 11.5 dBi with switchable linearly polarized (LP) and CP states.

## 2. MED Antenna: Design Methodology

This section discusses the design methodology for a novel wideband circularly polarized MED antenna. The antenna is designed, simulated, and optimized using ANSYS HFSS (Latest version 2025). The results from ANSYS HFSS [18] are then verified by simulating the optimized antenna using CST Microwave Studio (CST-MWS) [19]. A lumped-element equivalent circuit (EC) is developed using particle swarm optimization for the optimum MED antenna. Next, the final antenna is fabricated, and the measurements are compared against the simulation and EC results.

### 2.1. Antenna Design Procedure

**Figure 1:**
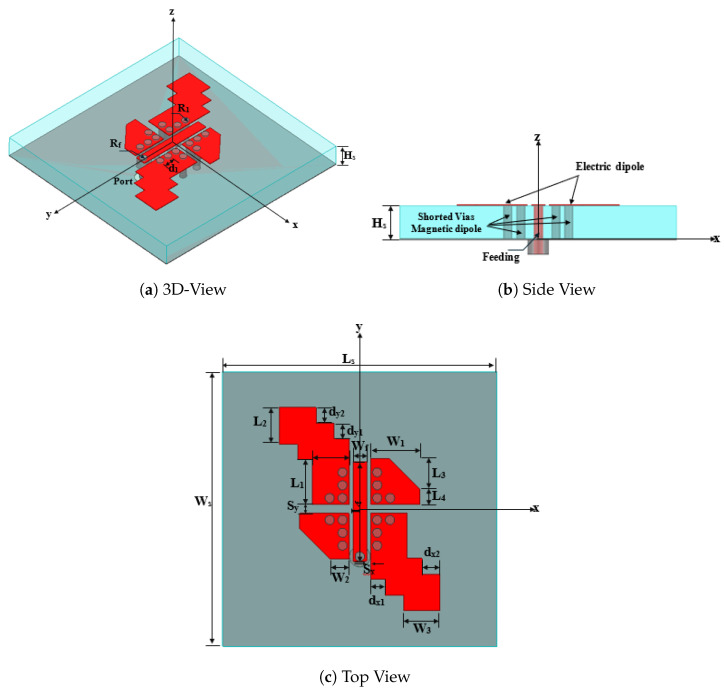
The geometry of a single-element wideband CP-MED antenna.

The MED antenna consists of three primary components: the electric dipole, the magnetic dipole, and the feeding element. The electric dipole consists of a pair of metal strips in a staircase design and a pair of rectangular strips with trimmed corners along the main diagonals. This configuration is introduced to excite two orthogonal degenerate modes ($TM_{10}$ and $TM_{01}$ equivalents) with equal amplitude and a $90^{\circ }$ phase difference, which is essential for generating CP radiation. Furthermore, four groups of shorted-via holes are used to implement the magnetic dipole part. In addition, the feeding element consists of an L-shaped probe connected to a 50 Ω SMA connector, which feeds the structure. The antenna was printed on a grounded Rogers 5880 substrate with a dielectric constant of 2.2, a loss tangent of 0.009, and a thickness of 1.5 mm. The final antenna geometry is shown in Figure 1.

**Figure 2:**
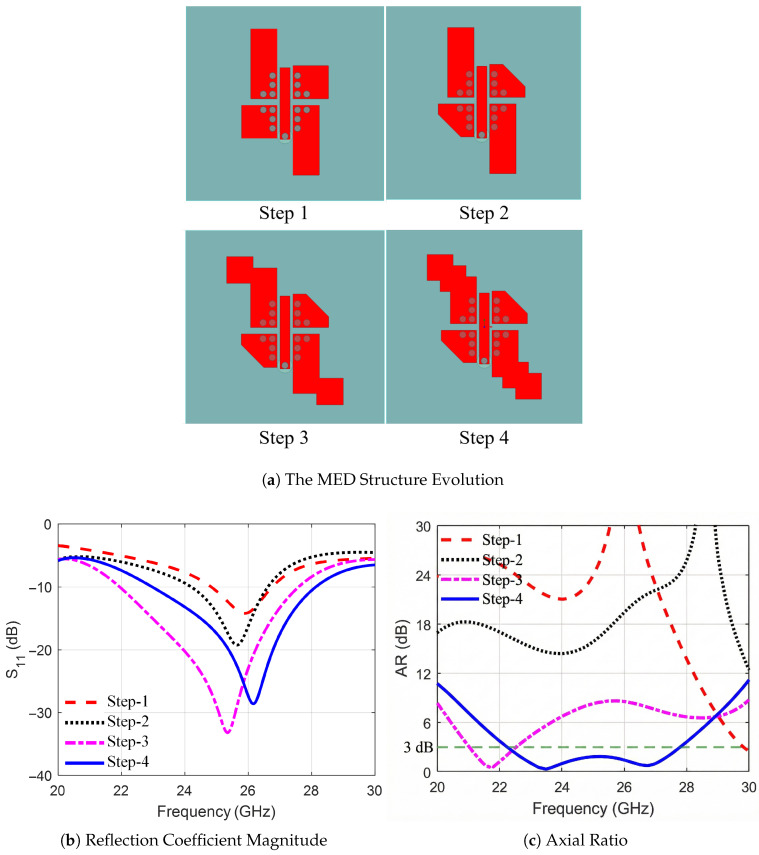
(**a**) The different design evolution steps for the MED antenna along with the corresponding (**b**) reflection coefficient magnitude and (**c**) axial ratio for each step.

**Figure 3:**
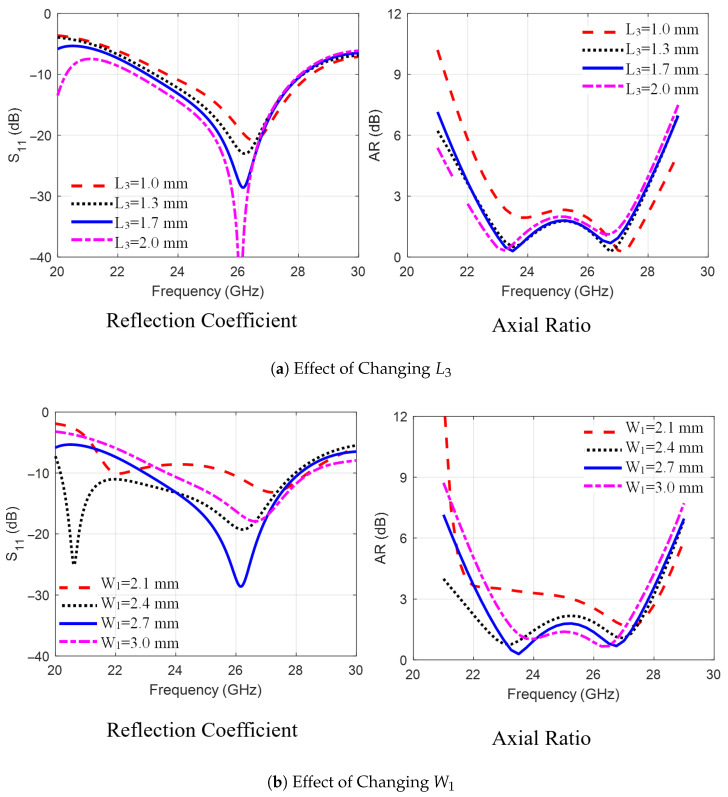
The effect of changing the MED antenna dimensions (**a**) $L_{3}$ and (**b**) $W_{1}$ on the radiation characteristics ($|S_{11}|$ and AR).

**Figure 4:**
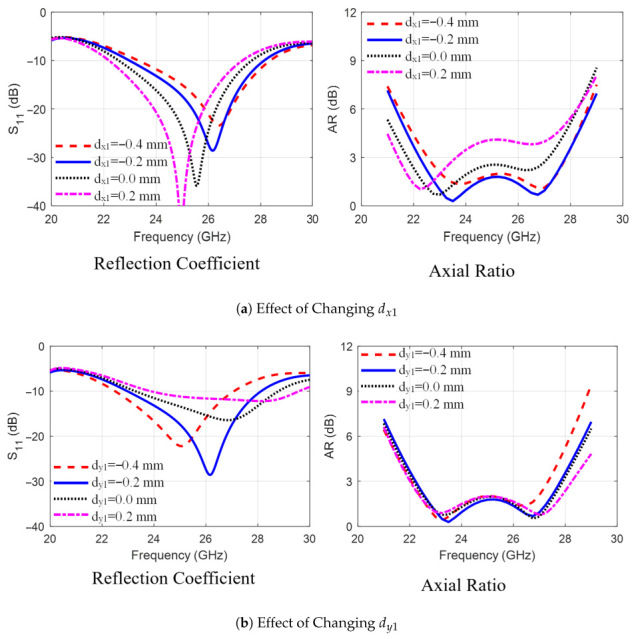
The effect of changing the MED antenna dimensions (**a**) $d_{x1}$ and (**b**) $d_{y1}$ on the radiation characteristics ($|S_{11}|$ and AR).

**Figure 5:**
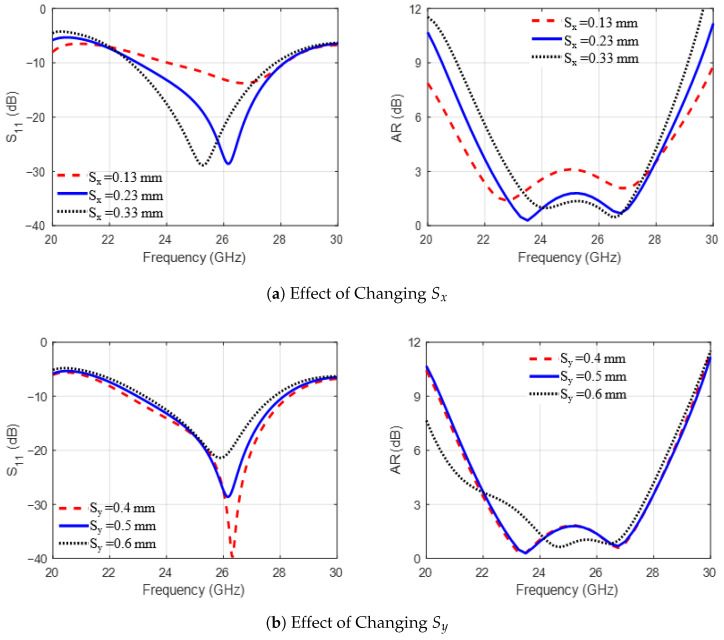
The effect of changing the MED antenna dimensions (**a**) $S_{x}$ and (**b**) $S_{y}$ on the radiation characteristics ($|S_{11}|$ and AR).

The antenna was designed in two stages. First, the MED antenna structure was obtained by following four steps, which are shown in Figure 2a. The objectives of this stage were: (1) The reflection coefficient magnitude $|S_{11}|$ should be less than −10 dB to achieve a wide-impedance matching bandwidth; (2) the axial ratio AR should be less than 3 dB to achieve wideband CP radiation; and finally, (3) the bandwidths for $|S_{11}|\;<-10$ dB and AR<3 dB must coincide to achieve a wideband performance in both impedance matching and CP. The antenna characteristics at different design evolution steps are shown in Figure 2b,c. In the second stage, a parametric study was carried out to investigate the effect of the different design dimensions, $L_{3}$, $W_{1}$, $d_{x1}$, $d_{y1}$, $S_{x}$, and $S_{y}$ on $|S_{11}|$ and AR bandwidths; the results of this study are shown in Figure 3, Figure 4 and Figure 5. The steps of the first stage are summarized as follows. In Step 1, two rectangular strips with shorted-via holes are designed to achieve asymmetrical displacement, resulting in a 1.9 GHz $|S_{11}|$ bandwidth with LP radiation (AR 20–30 dB). Next, in Step 2, small rectangular strips with trimmed corners are utilized to broaden the $|S_{11}|$ bandwidth to 2.4 GHz, with LP (AR 16–30 dB) and a CP (AR 3 dB) at 30 GHz. In Step 3 (single staircase), the step perturbs the current paths, shifting the resonance to lower frequencies and initiating CP from 21 GHz to 22.5 GHz by creating quadrature phase excitation.

Finally, the additional staircase in Step 4 further equalizes mode amplitudes, extending CP bandwidth to 5.6 GHz (22.23–27.83 GHz) by reducing AR sensitivity to frequency variations. The trimmed corners reduce cross-polarization by symmetrizing currents, while staircases create capacitive loading for quadrature modes, thereby broadening the AR bandwidth. This approach, unlike uniform patches, minimizes cross-polarization (≤−20 dB) and enhances CP bandwidth without increasing size.

The findings of the parametric study performed in the second stage are summarized next. As shown in Figure 3a, increasing the trimmed corner length $L_{3}$ from 1 mm to 2 mm increased the bandwidths of $|S_{11}|$ and AR by decreasing the lower frequency of the operating band. Next, as shown in Figure 3b, increasing the arm width $W_{1}$ at the trimmed corners from 2.1 mm to 3 mm impacts the matching and AR values in the frequency band, with the best results achieved at 2.7 mm. Next, Figure 4a,b show the effect of shifting the staircase step positions relative to the patch corner, $d_{x1}$ and $d_{y1}$, from −0.4 mm to 0.2 mm. It is observed that $d_{x1}$ affects the lower frequency limit while $d_{y1}$ shifts the upper limit of the operating band, with optimal values seen at $d_{x1}=d_{y1}=-0.2$ mm. Figure 5a,b investigate the effect of varying distance $S_{x}$ between the electric dipole arms and the L-feeder and changing the gap size $S_{y}$ between the stairs-strip and the rectangular strip with trimmed corners. As observed in Figure 5, changing $S_{x}$ from 0.13 mm to 0.33 mm and $S_{y}$ from 0.4 mm to 0.6 mm affects impedance matching and AR. This is due to changes in the induced current on the MED surface. From the parametric study, it can be concluded that the dimensions of the L-feeder, the rectangular strips, and the sets of via holes control the impedance-matching bandwidth of the proposed antenna. However, the CP radiation characteristics or AR are controlled via the staircase steps and trimmed corners of the rectangular strips. Furthermore, the distances between the L-feeder and the rectangular strip radiators, $S_{x}$ and $S_{y}$, affect both $|S_{11}|$ and the AR bandwidths. Additionally, it was observed during the study that small changes in other dimensions may deteriorate $|S_{11}|$ or AR bandwidths.

### 2.2. Antenna Final Design

**Figure 6:**
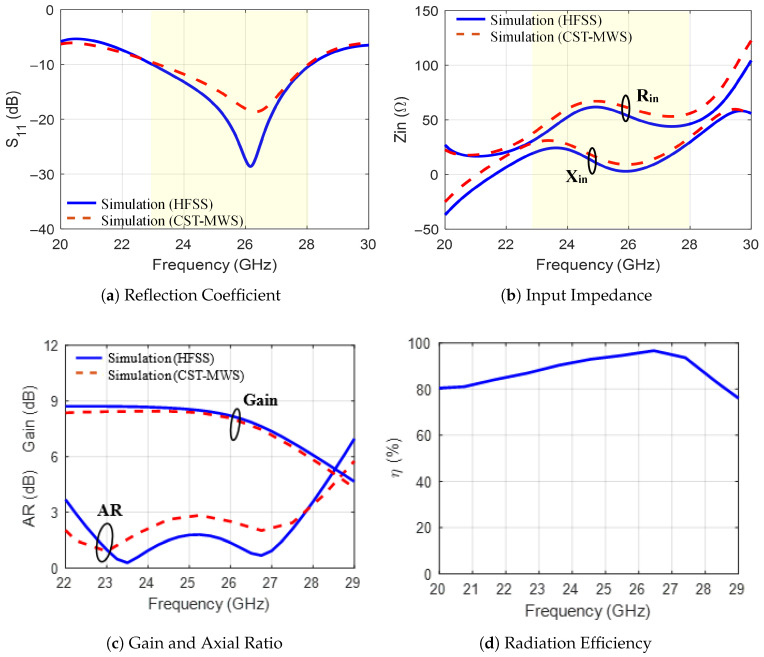
The simulated radiation characteristics of the final MED antenna. To verify the simulations, the results are shown for two software: HFSS and CST.

The optimized dimensions of the final MED antenna are given in Table 1. The optimized structure’s radiation characteristics ($|S_{11}|$ and AR) were simulated using ANSYS HFSS and CST Microwave Studio to verify the simulation results. The results are shown in Figure 6. The values of $|S_{11}|$ in Figure 6a indicate a well-matched antenna over the band from 22.97 GHz to 28.12 GHz (20.6% fractional bandwidth). The CP covers the band from 22.23 to 27.83 GHz with a peak gain of 8.8 dBi as shown in Figure 6c. The MED antenna introduces high radiation efficiency (≥90%) in the operating bandwidth, as shown in Figure 6d. Furthermore, since the results of both software are similar, the simulation setup of the proposed MED antenna is verified.

**Figure 7:**
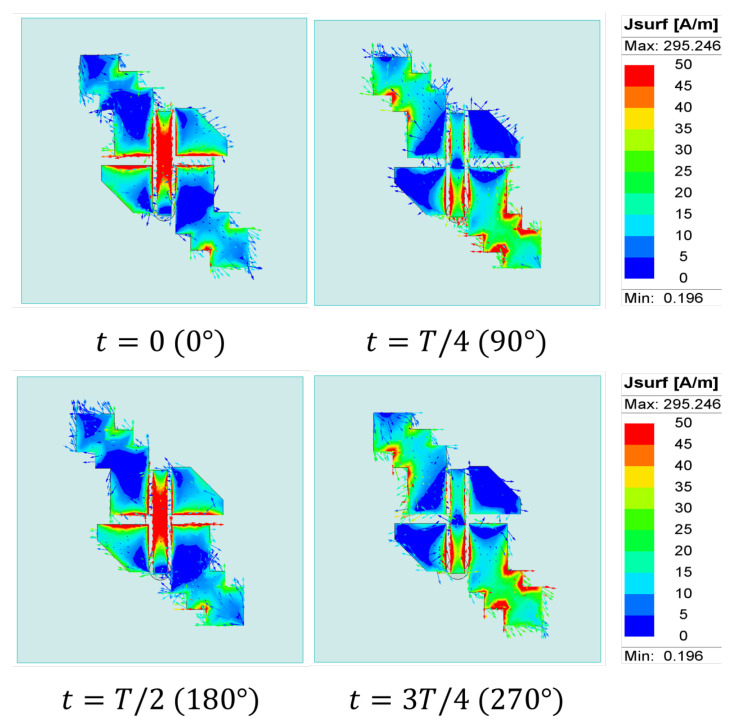
The surface current distribution of the wideband CP-MED antenna at 25 GHz.

**Figure 8:**
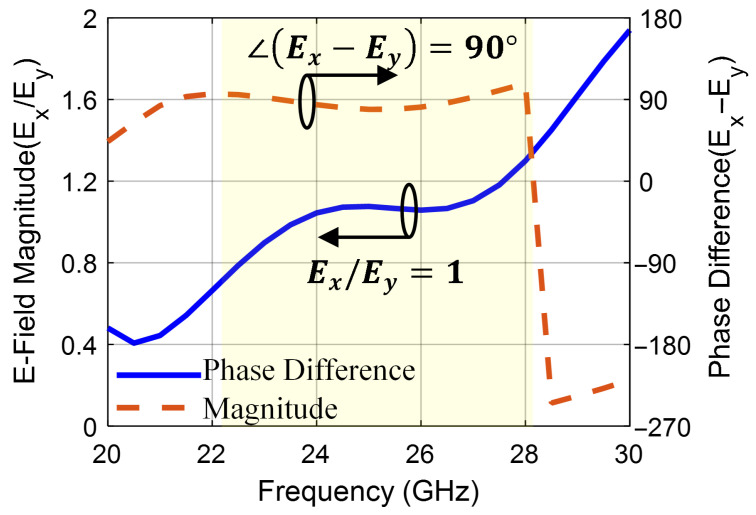
The magnitude and phase diagrams for the CP mechanism.

**Figure 9:**
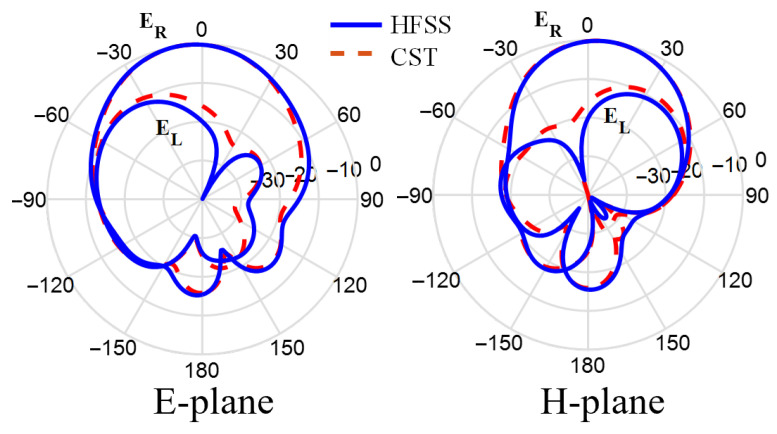
The radiation patterns at 25 GHz.

Next, Figure 7 shows the surface current distribution at 25 GHz for different time phases $0^{\circ }$, $90^{\circ }$, $180^{\circ }$, and $270^{\circ }$. It can be observed that the current rotates in an anticlockwise direction, generating RHCP field in the +z-direction. Two electric field components, $E_{x}$ and $E_{y}$, with equal amplitude and a $90^{\circ }$ phase shift, are generated from the electric dipole strips and magnetic dipole via holes as shown in Figure 8. Finally, the right-hand ($E_{R}$) and left-hand ($E_{L}$) electric field radiation patterns in the different planes are plotted in Figure 9. The symmetrical radiation patterns are radiated with a co-/cross-polar level ($E_{R}/E_{L}$) of 20 dB and half-power beamwidth (HPBW) of $78^{\circ }$.

### 2.3. Antenna Equivalent Circuit

**Figure 10:**
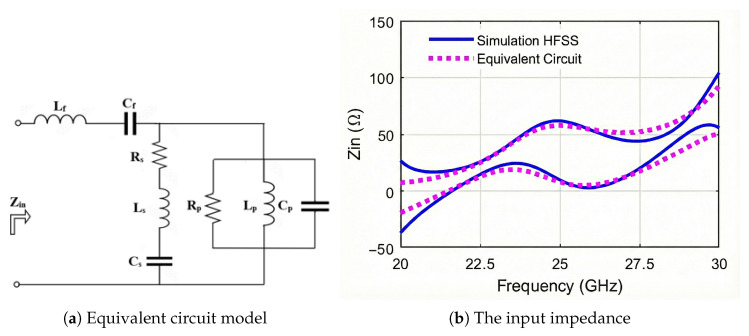
(**a**) The equivalent circuit model of the MED antenna, and (**b**) the simulated (HFSS) input impedance compared to the EC results.

The equivalent circuit (EC) model characterizes the antenna’s electrical behavior as a network of lumped elements, which includes resistors, inductors, and capacitors [20,21,22]. The EC effectively models the resonant and impedance properties that govern the antenna’s interaction with electromagnetic waves. The EC of the proposed wideband MED antenna is presented in Figure 10a. The lumped-element EC accurately equivalent the MED physical structure by modeling the electric dipole as a series RLC branch ($C_{s}$ for staircase capacitance, $L_{s}$ for strip inductance, $R_{s}$ for losses) and the magnetic dipole as parallel RLC branches, ($R_{p}$, $L_{p}$, and $C_{p}$) for shorted via holes, with the L-probe inductive and capacitive effects modeled by series circuit ($L_{f}$ and $C_{f}$) aiding matching to 50 Ω with the source. The EC input impedance of the MED antenna is given by:

(1) $$\begin{array}{cc} Z_{\mathrm{in}} & =j\left(\omega L_{f}-\frac{1}{\omega C_{f}}\right)\\ \; & \;+\frac{1}{\frac{1}{R_{p}}+j\left(\omega C_{p}-\frac{1}{\omega L_{p}}\right)+\frac{1}{R_{s}+j\left(\omega L_{s}-\frac{1}{\omega C_{s}}\right)}} \end{array}$$

The superposition of the electric and magnetic dipoles’ resonance frequencies leads to the MED antenna’s wideband characteristics [23]. Particle Swarm Optimization (PSO) is employed to determine the EC elements by iteratively adjusting their values within a defined search space to minimize a fitness function, as discussed in [22]. The fitness function is the mean square error (MSE) between the simulated input impedance (HFSS), $Z_{in}^{HFSS}\left(f_{n}\right)$ and the impedance calculated using the EC model, $Z_{in}^{PSO}\left(f_{n}\right)$, over a range of frequency points, $N_{f}$. The fitness function *F* is defined by [20]:

(2) F=1Nf∑n=1Nf[ReZinHFSS(fn)−ReZinPSO(fn)2+ImZinHFSS(fn)−ImZinPSO(fn)2].

Table 2 presents the estimated values of the EC elements, obtained by PSO via employing 26 particles at 1001 frequency points. Figure 10 compares the simulated input impedance with that calculated using the EC model. The proposed EC model effectively characterizes the MED antenna across a wide frequency band, achieving a mean square error (MSE) of 0.22% in the resistance component and 3.09% in the reactance component relative to the simulation results.

The EC guides the design by enabling rapid parametric sweeps. For example, increasing the number of staircase steps correlated with higher *C* values, predicting AR bandwidth extension over the operating frequency band. This analytical tool complements HFSS/CST simulation to optimize dimensions prior to fabrication, ensuring wideband CP without the need for empirical trials.

### 2.4. Antenna Fabrication Results

**Figure 11:**
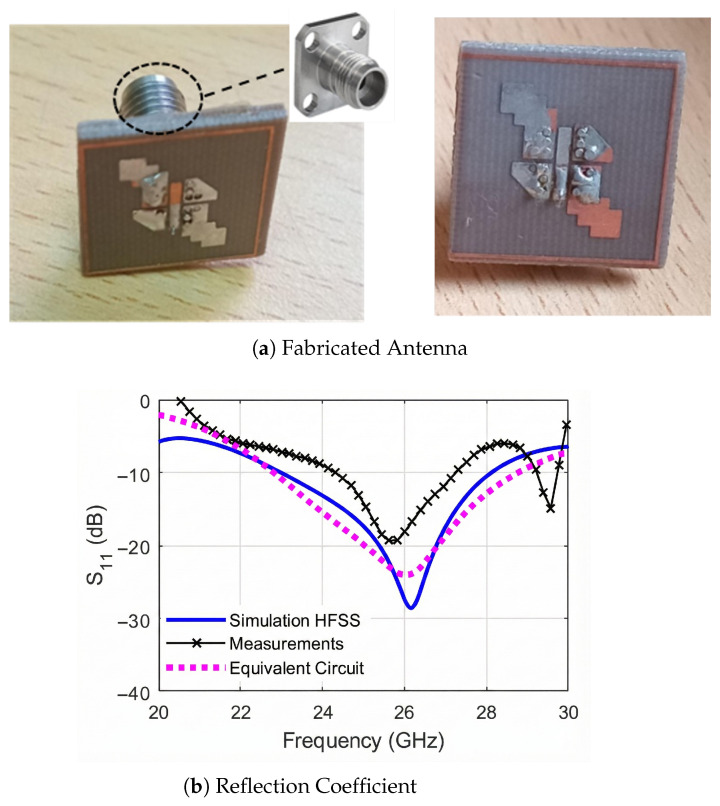
(**a**) The fabricated MED antenna prototype, and (**b**) the reflection coefficient measurements compared to the simulation and EC results.

A prototype of the proposed MED antenna is fabricated on a Rogers 5880 substrate with dimensions $1.28\lambda_{0}\times 1.28\lambda_{0}\times 0.128\lambda_{0}$, where $\lambda_{0}$ is calculated at 25.5 GHz. The fabricated antenna is shown in Figure 11a. The prototype is fed using a 50Ω 2.92 mm connector. The magnitude of the reflection coefficient $S_{11}$ of the prototype is measured using a calibrated Anritsu MS46322A (Anritsu, Kanagawa, Japan) vector network analyzer. The measurement results are plotted in Figure 11b and are compared with the simulation and EC results. The measured $|S_{11}|$ fractional bandwidth is 12.6% (24.13 GHz to 27.32 GHz). The differences between the measured and simulated results are due to the soldering effect and the losses from the connector and cable. Throughout the paper, the far-field radiation characteristics, including gain, AR, and radiation patterns, are simulated using ANSYS HFSS and CST Microwave Studio, as our laboratory currently lacks the necessary measurement equipment to conduct experimental validations.

Furthermore, a comparison between the proposed MED antenna and work reported in the literature is presented in Table 3. Although the size of the proposed MED is larger than that in [24,25], it prioritizes an AR bandwidth of 21.9% compared to 15.1% in [24] and 7.3% in [25] with a gain enhancement by 1.5 dBi. The increased size accommodates ground slots for ≥23 dB isolation, reducing mutual coupling by 5 dB over without parasitic elements, enhancing suitability for 5G massive MIMO.

## 3. MIMO-CP Antenna: Design Methodology

This section first discusses the design methodology for a 1×2 MIMO antenna using the optimized MED element from Section 2. Furthermore, a numerical investigation is performed using two MED elements on a MIMO-CP antenna for 5G applications. The investigated parameters are the antennas’ configuration with respect to each other, the separation between them, and the introduction of a cutting slot in the ground between the antennas. Next, based on the 1×2 MIMO antenna investigation, the design is expanded to a 2×2 MIMO-CP antenna.

### 3.1. Antennas’ Configuration

**Figure 12:**
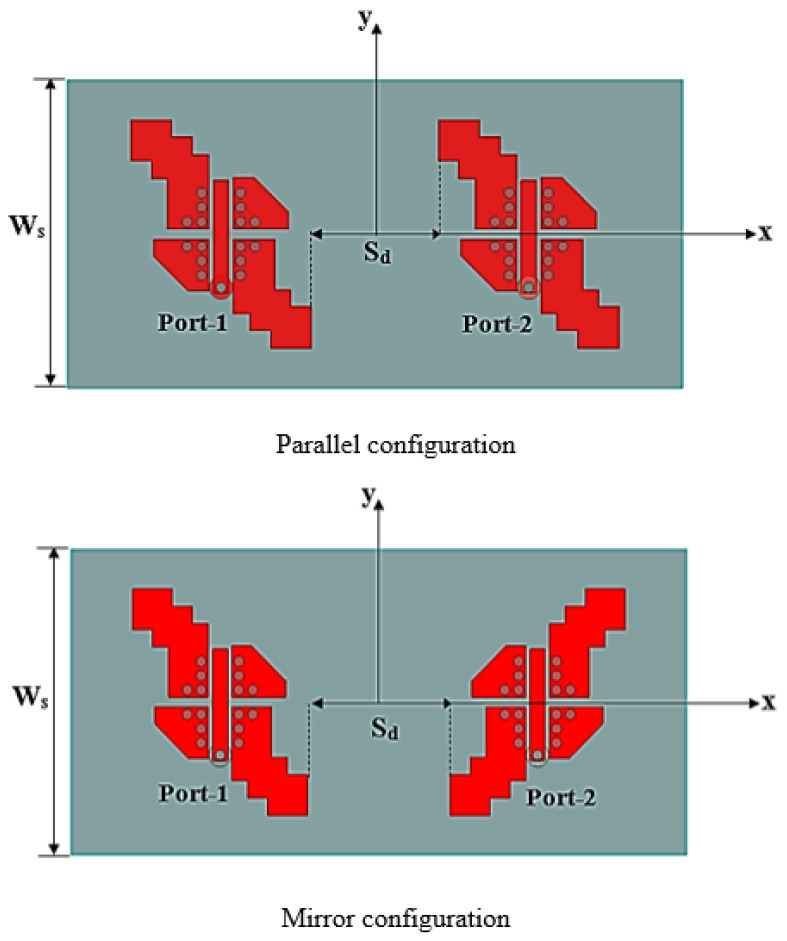
The 1×2 MIMO-CP Antenna configurations.

**Figure 13:**
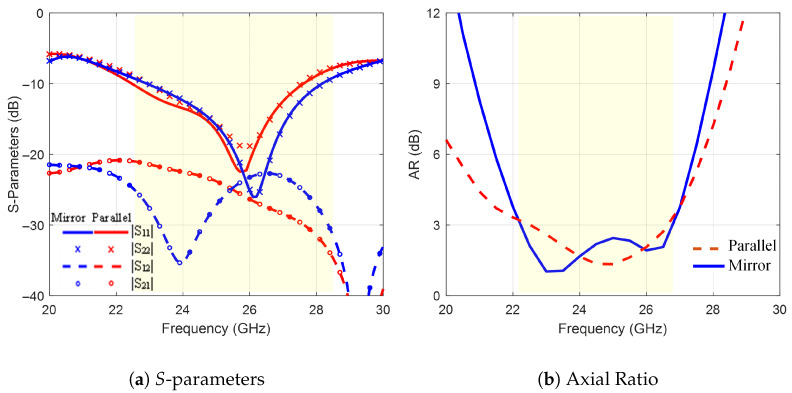
The 1×2 MIMO-CP Antenna configurations: (**a**) *S*-parameters and (**b**) axial ratio.

The performance of the 1×2 MIMO antenna is studied under two configurations. The single antenna is either duplicated in a parallel or a mirror configuration, as shown in Figure 12. Here, the MIMO-CP antenna size is 15×30×1.5 mm^3^ with an interconnected grounded substrate to maintain uniform voltage levels for the entire antenna system. Figure 13a,b compare the two MIMO configurations based on $|S_{11}|$, $|S_{22}|$, $|S_{12}|$, $|S_{21}|$, and AR. The parallel configuration introduces non-coherent reflection coefficients $|S_{11}|$ and $|S_{22}|$ at the two ports with the elements isolation ($|S_{12}|$ and $|S_{21}|$) below −20 dB. The mirror configuration introduces coherent reflection coefficients with a better match (−26 dB) and good isolation, ranging from −23 dB to −36 dB at 24 GHz. Furthermore, the mirror configuration extends the AR bandwidth to 4.35 GHz, compared to 4.12 GHz for the parallel configuration.

**Figure 14:**
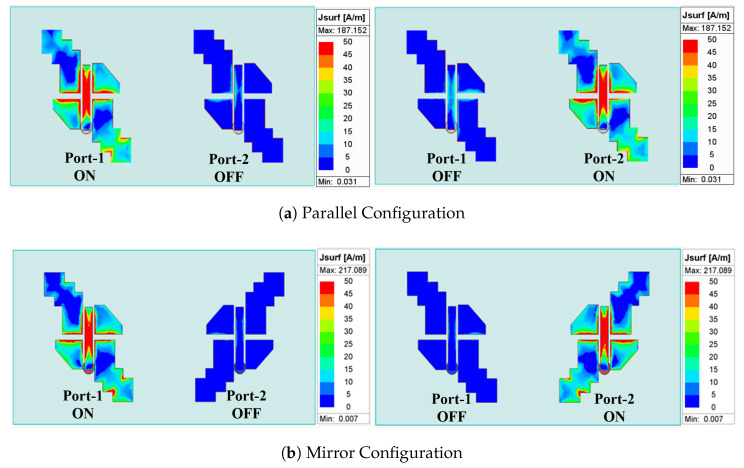
The current distribution for each port excited at 25 GHz for (**a**) parallel and (**b**) mirror configurations.

The surface current distributions at 25 GHz for both configurations at different port excitations are shown in Figure 14. In these simulations, when a port is excited (ON), the other port (OFF) is terminated with a matched load. From Figure 14b, it can be observed that the surface current is maximum on the excited element (ON), while it is zero on the other element (OFF) for the mirror configuration; thus, high isolation is achieved between elements. However, for the parallel configuration in Figure 14a, there is a small induced surface current on the L-feeder of the OFF element.

In conclusion, the polarization diversity in the mirror configuration enhances the isolation between elements and improves impedance matching, which aligns with the AR bandwidth. Thus, in the next sections, only the mirror configuration is considered.

### 3.2. Antennas’ Separation

**Figure 15:**
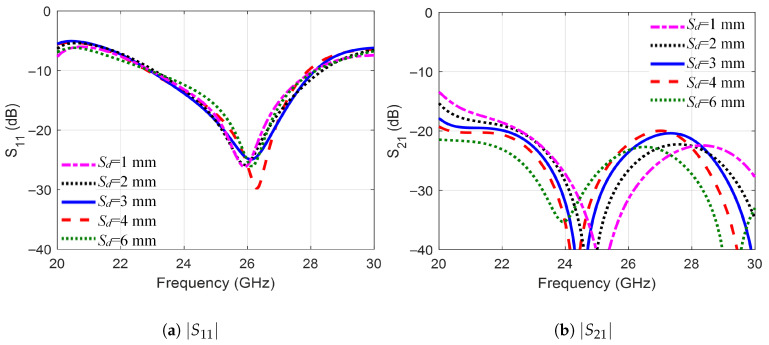
The variation of the *S*-parameters versus frequency for the mirror configuration for different $S_{d}$.

A parametric study investigated the effect of changing the separation between the individual elements in the MIMO mirror configuration. Figure 15 shows the effect of changing the inter-element spacing, $S_{d}$, from 1 mm to 6 mm on $|S_{11}|$ and $|S_{21}|$. As the figure shows, the closely placed elements still have good isolation ($|S_{21}|\;<-23$ dB), with better isolation achieved at the high-frequency band. Thus, $S_{d}=2$ mm was selected to reduce the size of the whole structure.

### 3.3. Ground Slot

Next, a cutting slot is centrally etched on the ground plane between the individual elements to compensate for the effect of closely spaced elements in the MIMO-CP structure. The cutting slots eliminate the current leakage between the elements, improving isolation and increasing the AR bandwidth.

**Figure 16:**
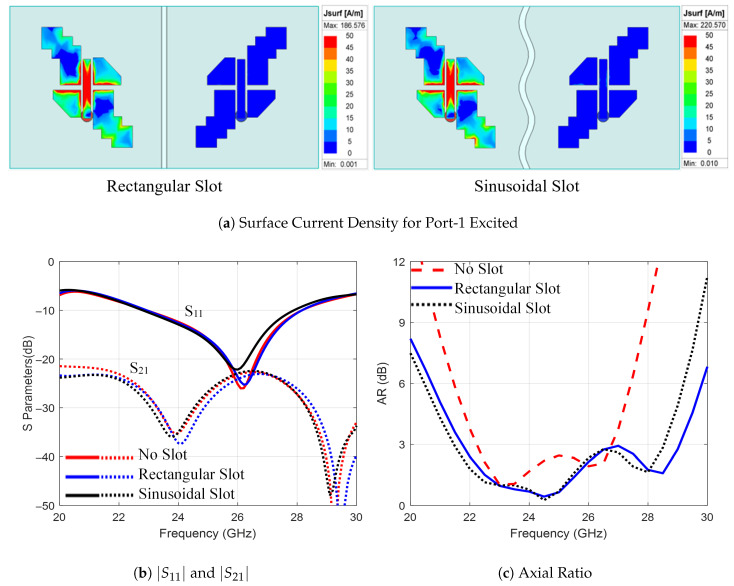
The 1×2 MIMO with different shapes of the ground slot.

Two different-shaped slots are investigated: a rectangular slot and a sinusoidal slot. Each slot had a length of 15 mm and a width of 0.6 mm. Here, the MIMO mirror configuration with $S_{d}=2$ mm was considered. The surface current density for different ports excited at 25 GHz is shown in Figure 16, along with the S-parameters and the AR results. As shown in Figure 16b, the bandwidth is improved to 5.3 GHz for rectangular slots and 5.1 GHz for sinusoidal slots. Moreover, as shown in Figure 16c, the AR bandwidth is 7.3 GHz and 7 GHz for rectangular and sinusoidal slots, respectively. In conclusion, the rectangular slot is considered for the MIMO configuration.

**Figure 17:**
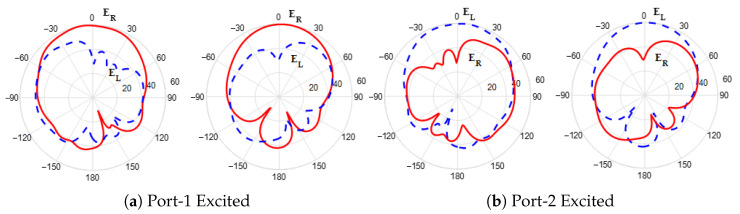
The 2D electric field radiation pattern for different port excitation at 25 GHz.

**Figure 18:**
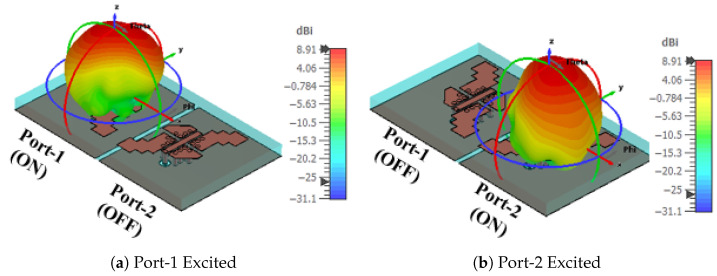
The 3D gain radiation pattern for 1×2 MIMO system at different port excitation at 25 GHz.

Next, the 2D electric field components $E_{R}$ and $E_{L}$ radiation patterns for ports 1 and 2 at 25 GHz for the rectangular slot are shown in Figure 17. Port-1 excitation results in the dominance of the RHCP field component. In contrast, port-2 excitation in the broadside direction leads to the dominance of the LHCP component. Moreover, the results show that the radiation pattern of each element was not affected by the MIMO configuration, which is due to the high isolation between the elements. Figure 18 shows the 3D gain pattern radiated by each element of a 1×2 MIMO system operating at 25 GHz. The elements exhibit identical broadside radiation patterns, achieving a peak gain of 8.9 dBi per element, which is attributed to the high isolation between them.

### 3.4. A 2×2 MIMO

**Figure 19:**
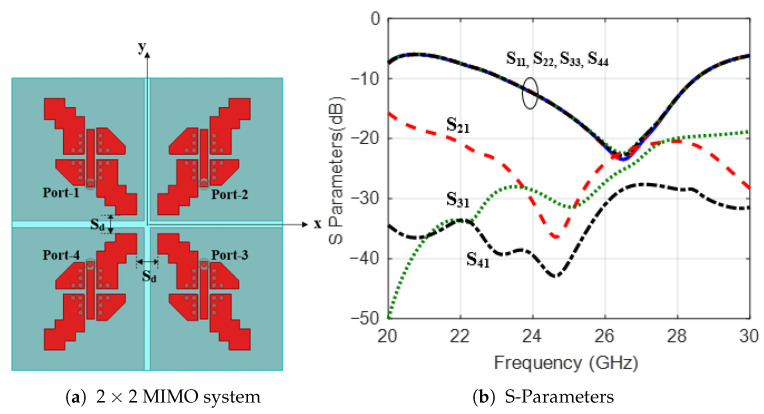
The arrangement of 2×2 MIMO system and the corresponding S-parameters.

**Figure 20:**
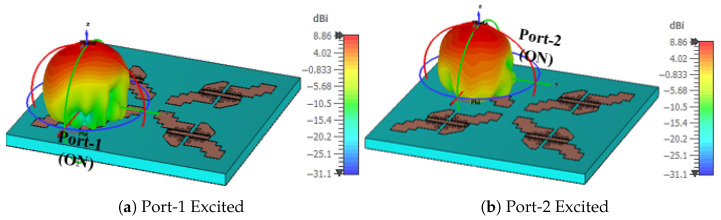

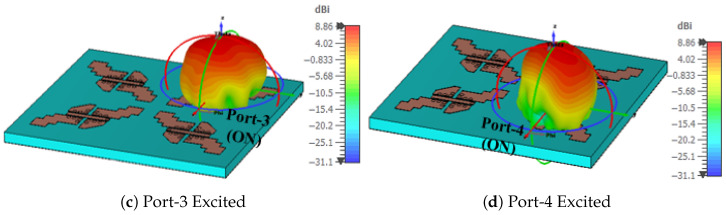
The 3D gain radiation pattern for 2×2 MIMO system at different port excitation at 25 GHz.

Based on the results from the previous sections, a 2×2 MIMO system with four MED antenna elements is investigated and is shown in Figure 19a. The MED elements are separated by $S_{d}=2$ mm to maintain high isolation between them. Figure 19b shows the response of the S parameters for the 2×2 MIMO system with ($|S_{11}|\;<-10$ dB) over the frequency band from 22 GHz to 28 GHz, with mutual coupling between ports maintained below ($|S_{12}|\;<-20$ dB). The 3D gain patterns for one single port (ON) at 25 GHz are shown in Figure 20. An identical broadside gain pattern with a peak gain of 8.9 dBi is radiated from each element in the 4-element MIMO system.

**Figure 21:**
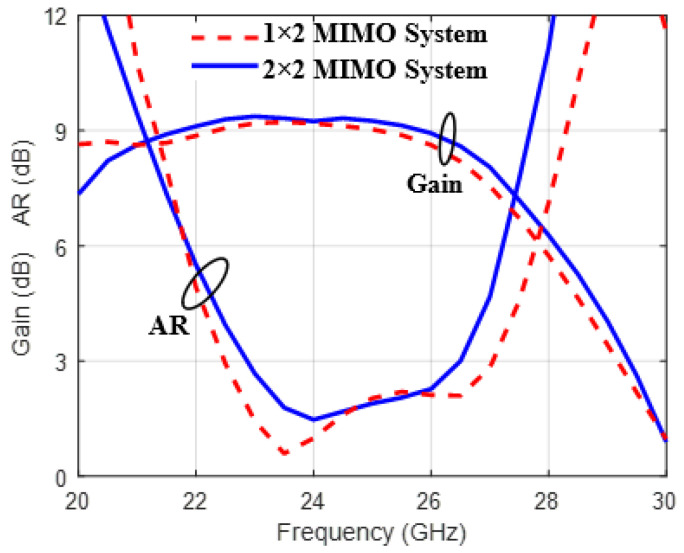
The gain and axial ratio of 1×2 and 2×2 MIMO systems.

Figure 21 shows the gain frequency response and the axial ratio of the 1×2 and 2×2 MIMO systems. Over the operating bandwidth, the gain varies from 8 dBi to 8.9 dBi, with AR bandwidth extending from 22.6 GHz to 27.1 GHz for a 1×2 MIMO system and from 23 GHz to 26.5 GHz for a 2×2 MIMO system. In conclusion, expanding the MIMO to a 2×2 design improved performance without affecting individual elements.

## 4. MIMO Diversity Performance Analysis

In this section, the MIMO diversity performance analysis is investigated for the 2×2 MIMO antenna system with optimized elements using different parameters. The considered MIMO is the mirror configuration with a ground rectangular slot and an $S_{d}=2$ mm inter-element separation. The study is conducted to ensure the validity of the MIMO system within the performance metric threshold limits introduced in the literature [27,28]. First, the envelope correlation coefficient (ECC) is evaluated, which shows the efficiency of the communication channel isolation. The ECC can be computed using the scattering (S) parameters for high-efficiency antennas, or using the far-field radiation pattern for low-efficiency antennas to account for losses [27].

Utilizing the S-parameters, the ECC between each pair of antenna elements can be calculated as

(3) $$ECC_{ij}=\frac{{\left|S_{ii}^{*}S_{ij}+S_{ji}^{*}S_{jj}\right|}^{2}}{\left(1-{\left|S_{ii}\right|}^{2}-{\left|S_{ji}\right|}^{2}\right)\left(1-{\left|S_{jj}\right|}^{2}-{\left|S_{ij}\right|}^{2}\right)}.$$

Here {i,j}={1,2,3,4}, and “ * ” denotes the complex conjugate of the S-parameter. $S_{ii}$ and $S_{jj}$ are the reflection coefficients at ports *i* and *j*, respectively, and $S_{ij}$ and $S_{ji}$ are the transmission coefficients between ports *i* and *j*.

Furthermore, by using the radiation pattern, the ECC can be evaluated as

(4) $$ECC_{ij}=\frac{{\left|\int 4\pi \left[E_{i}\left(\theta ,\phi \right)\cdot {\left[E_{j}\left(\theta ,\phi \right)\right]}^{*}\right]d\Omega \right|}^{2}}{\int 4\pi {\left|E_{i}\left(\theta ,\phi \right)\right|}^{2}d\Omega \cdot \int 4\pi {\left|E_{j}\left(\theta ,\phi \right)\right|}^{2}d\Omega }.$$

Here $E_{\left\{i,j\right\}}\left(\theta ,\phi \right)$ is the 3D radiation pattern field with excitation at port {i,j}, “·” denotes the Hermitian product, and Ω is the solid angle.

**Figure 22:**
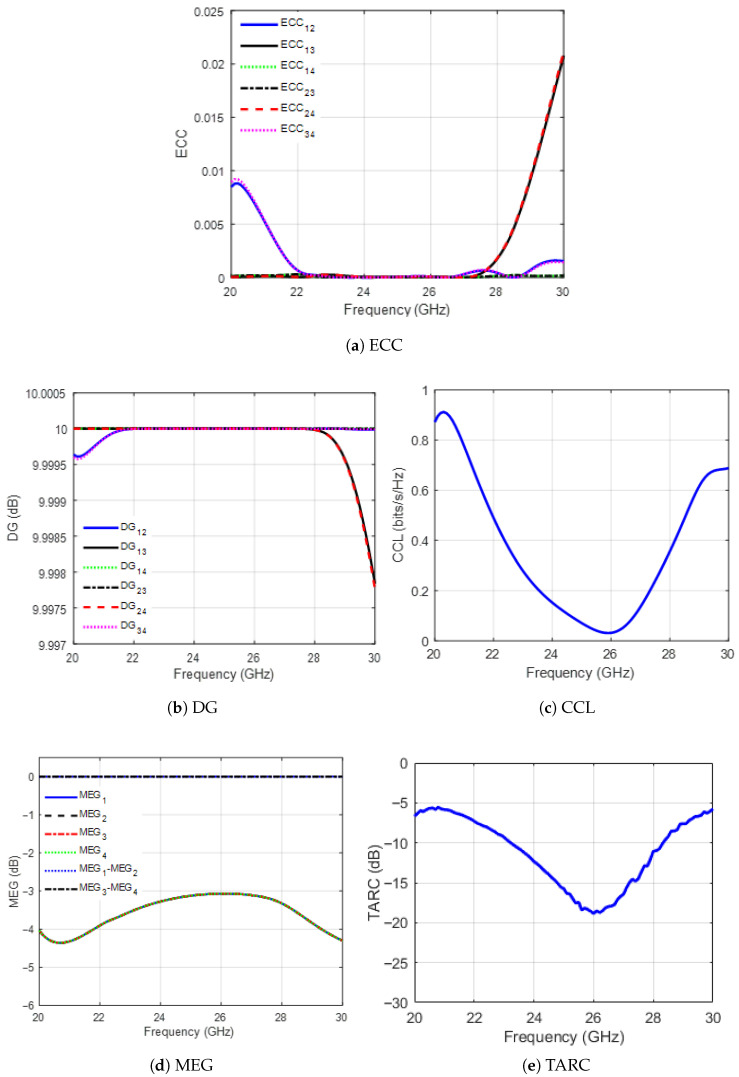
The 2×2 MIMO-CP MED antenna system diversity performance analysis parameters.

Furthermore, an ideal MIMO system has a zero ECC value; however, ECC < 0.5 is acceptable [27]. In a 4-element MIMO system, the ECC should be calculated for each pair of elements (for example, {1,2}, {1,3}, {1,4}, {2,3}, {2,4}, {3,4}), with the maximum ECC generally reported as a conservative measure of the system’s correlation. The ECC result for the 2×2 MIMO-CP MED antenna system is shown in Figure 22a. Within the antenna bandwidth, the ECC value is less than $1\times 10^{-3}$ over the band from 22 GHz to 28 GHz, which satisfies the requirement and indicates a low correlation between the antennas. Consequently, the mutual coupling between the elements is low, which is desirable.

Next, diversity gain (DG) is evaluated. DG is the power transmission loss due to the employment of the diversity scheme in the MIMO systems [28]. It is calculated as

(5) $$DG_{ij}=10\sqrt{1-|ECC_{ij}{|}^{2}}.$$

Based on the ECC results in Figure 22a, the value of DG for each pair of elements of the proposed MIMO is approximately 10 dB within the system bandwidth, which agrees with the required threshold to ensure good diversity performance of the MIMO system. The DG values are shown in Figure 22b.

To measure the maximum data transmitted across a communication channel without losses, the channel capacity loss (CCL) is calculated. The CCL for 4-port MIMO is calculated as follows [27]:

(6) $$\mathrm{CCL}=-\log_{2}\det\left(X^{R}\right),$$

where

(7) $$X^{R}=\left[\begin{array}{cccc} X_{11} & X_{12} & X_{13} & X_{14}\\ X_{21} & X_{22} & X_{23} & X_{24}\\ X_{31} & X_{32} & X_{33} & X_{34}\\ X_{41} & X_{42} & X_{43} & X_{44} \end{array}\right],$$

(8) $$X_{ii}=1-\sum_{n=1}^{4}{\left|S_{in}\right|}^{2},$$

and

(9) $$X_{ij}=-\left(S_{ii}^{*}S_{ij}+S_{ji}^{*}S_{jj}\right).$$

The CCL results are shown in Figure 22c. Within the MIMO bandwidth, the CCL value is less than 0.35 bits/s/Hz. This result is acceptable since it is less than the threshold value of 0.5 bits/s/Hz required for industrial applications [28].

The next calculated parameter is the Mean effective gain (MEG) of the MIMO antenna. MEG is the ratio of the MIMO antenna’s received power to the isotropic antenna’s received power. MEG is calculated for each port of the system as [27]

(10) $$\begin{array}{c} {\mathrm{MEG}}_{i}=\eta_{i}\left(1-\sum_{j=1}^{4}{\left|S_{ij}\right|}^{2}\right). \end{array}$$

Here $\eta_{i}$ is the radiation efficiency of the *i*th antenna. Furthermore, a MIMO antenna’s performance improves when the ratios of the MEG1/MEG2 and MEG3/MEG4 are less than 3 dB [28]. Moreover, Figure 22d shows the plots of MEG1, MEG2, MEG3, MEG4, MEG1/MEG2, and MEG3/MEG4. In this figure, the values of the MEG1/MEG2 and MEG3/MEG4 are 0 dB within the operating band.

The last calculated parameter is the total active reflection coefficient (TARC), which describes the changes in self and mutual impedance of the individual and adjacent elements. In MIMO systems, the allowable value for TARC should be less than −10 dB. TARC is calculated as [27]

(11) $$\mathrm{TARC}=\frac{\sqrt{\sum_{i=1}^{4}{\left|S_{ii}\right|}^{2}+\sum_{n=2}^{4}{\left|S_{in}e^{j\Theta_{n-1}}\right|}^{2}}}{2}.$$

Here Θ is the excitation phase difference between the two ports. From the results shown in Figure 22e, the TARC values are less than −10 dB within the desired band; thus, the requirement is satisfied.

In conclusion, the performance parameters of the MED MIMO system are presented and compared with those reported in the literature in Table 4. The proposed antenna meets the requirements of MIMO systems with high-gain and efficient performance with consistent impedance matching and CP bandwidths.

## 5. Wideband Sequential MED Antenna Arrays

**Figure 23:**
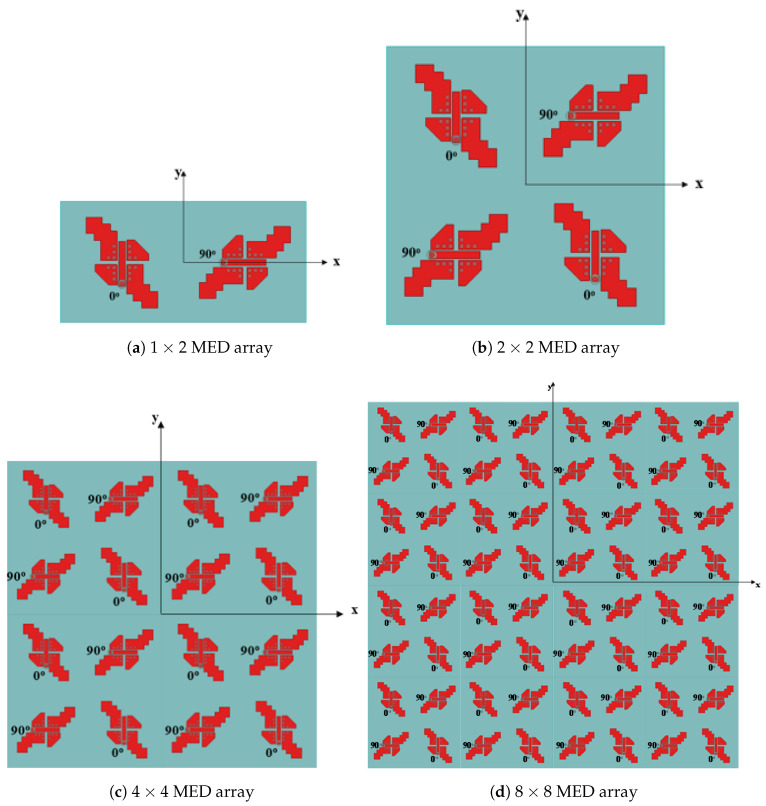
The arrangements of CP sequential MED antenna arrays.

**Figure 24:**
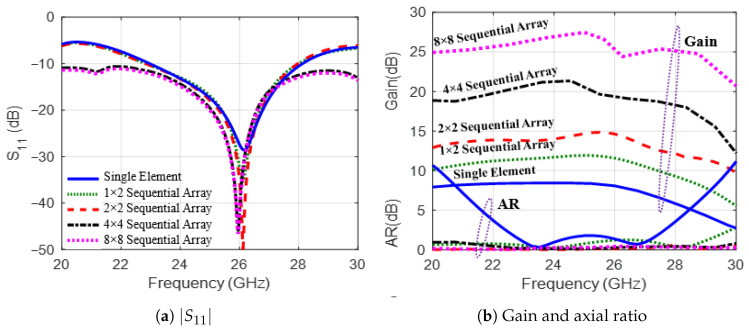
The radiation characteristics of different arrangements of CP sequential MED antenna arrays.

The radiation characteristics of the MED antenna are significantly improved by implementing a sequential array configuration [32,33]. It employs a sequential array of elements characterized by a $90^{\circ }$ orientation, rotation angle, and a $90^{\circ }$ phase difference between adjacent elements. For example, in [34], the authors designed an 8×8 SIW-MED antenna array for CP radiation over the frequency band from 23.3–30.8 GHz and AR bandwidth of 27.8% (23.2–30.8 GHz) with a peak gain of 20.2 dBi. Figure 23 shows various sequential array arrangements, 1×2, 2×2, 4×4, and 8×8 MED element configurations, to evaluate their impact on antenna performance. The frequency responses of the reflection coefficients $|S_{11}|$, gain, and AR for these different array arrangements are presented in Figure 24.

**Figure 25:**
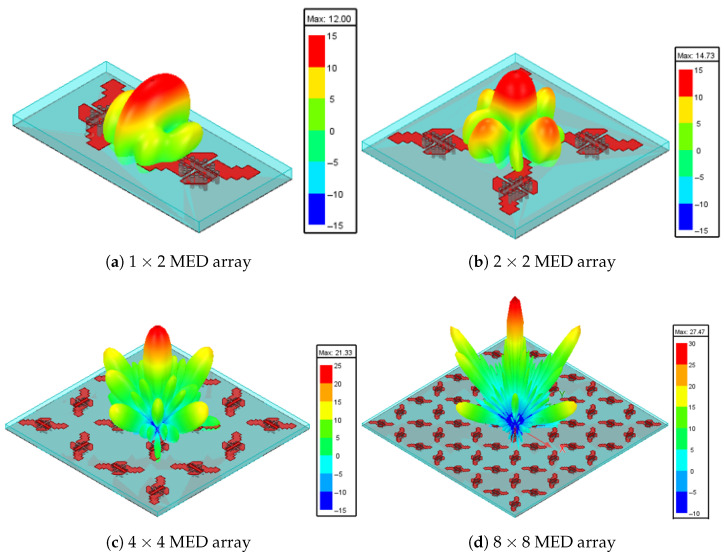
The 3D gain patterns of different arrangements of CP sequential MED antenna arrays.

The single element achieves a minimum $|S_{11}|$ of approximately −25 dB around 25 GHz, with a fractional bandwidth of 20.6% as shown in Figure 24a. The sequential array improves the impedance matching with a minimum of −30 dB for the 1×2 array, and −40 dB for the 2×2 array. Furthermore, the 4×4 and 8×8 arrays exhibit the best impedance matching with a minimum $|S_{11}|$ of −55 dB at 25 GHz, corresponding to a significantly enhanced fractional bandwidth of 40%. As shown in Figure 24b, the gain for the single element peaks at around 8 dBi. Furthermore, the sequential arrays demonstrate progressive improvement: the 1×2 array achieves a peak gain of 12 dBi, the 2×2 array reaches 14.7 dBi, the 4×4 array attains a 21.3 dBi gain, and the 8×8 array enhances the gain to 27.47 dBi with all configurations maintaining stability across the operating bandwidth. Moreover, incorporating a $90^{\circ }$ sequential phase shift between array elements significantly improves the CP bandwidth, maintaining an AR≤1 dB and stable gain across the entire frequency range from 20 GHz to 30 GHz. Figure 25 illustrates the 3D gain patterns at 25 GHz for different sequential arrays. The broadside radiation characteristic has a peak gain of 12 dBi, 14.7 dBi, 21.3 dBi, and 27.47 dBi for the 1×2, 2×2, 4×4, and 8×8 MED array configurations, respectively. The cumulative effect of increasing the array size and the sequential phase improves the directivity of the main lobe and reduces the side lobes.

In [35], a 4×4 MED array achieves a peak gain of 19.8 dBi with a wide-impedance bandwidth (24.5–40.8 GHz) and CP bandwidth (27.5–43 GHz). However, its multilayer design with five substrates increases fabrication complexity. In contrast, the proposed sequential 4×4 MED array in this work uses a single-layer Rogers5880 substrate with staircase dipoles, achieving a higher gain of 22 dBi, consistent impedance bandwidth, and an AR≤1 dB over 20–30 GHz, which allows easier integration for 5G systems.

## 6. Conclusions

A wideband circularly polarized novel MED antenna is designed, optimized, and implemented for 5G mmWave applications. The antenna features a proximity-coupled L-feeder, an electric dipole composed of diagonally placed rectangular staircase strips and trimmed corner strips, and a magnetic dipole made of sets of via holes. To achieve wideband impedance matching and CP bandwidths, the design evolution is investigated through a four-step process and a parametric study. Next, various MIMO systems utilizing the proposed MED antenna are designed. The effects of the elements’ arrangement, the separation between them, and the inclusion of a ground slot are studied. Furthermore, a MIMO diversity performance analysis is conducted for the optimum configuration of a 2×2 MIMO antenna system. The results show that the performance metrics fulfill the requirements within the desired bandwidth.

Finally, MED arrays are investigated, achieving wideband performance through the implementation of sequential array configurations. Employing sequential rotation and a $90^{\circ }$ phase shift between MED array elements enhances impedance matching and CP bandwidths over the band from 20 GHz to 30 GHz. A high gain of 27.47 dBi with AR≤1 dB is achieved using an arrangement of 8×8 array. All reported AR, radiation pattern, and gain results are simulation-based, due to the unavailability of far-field measurement facilities in this study. In future work, we intend to conduct experimental far-field measurements to validate these results and optimize the design for practical applications.

## Figures and Tables

**Table 1:** The optimized dimensions of the final MED antenna.

Parameter	Value (mm)	Parameter	Value (mm)	Parameter	Value (mm)
$L_{s}$	15	$W_{s}$	15	$d_{x1}$	0.80
$L_{1}$	2.45	$W_{1}$	2.7	$d_{x2}$	1.0
$L_{2}$	2.0	$W_{2}$	1.0	$d_{y1}$	0.85
$L_{3}$	1.7	$W_{3}$	2.0	$d_{y2}$	0.85
$L_{4}$	0.8	$W_{f}$	0.75	$d_{1}$	1.20
$L_{f}$	5.45	$R_{f}$	0.30	$R_{1}$	0.30
$H_{s}$	1.5	$S_{x}$	0.23	$S_{y}$	0.50

**Table 2:** Estimated values of the equivalent circuit elements for the MED antenna obtained using PSO.

Variable	PSO Variation Range	Final Value
Number of iterations *n*	1.0–10,000	6000
$L_{f}$ (H)$\;\times \;10^{-11}$	1–90	24.3
$C_{f}$ (F)$\;\times \;10^{-15}$	50–100	88.7
$R_{s}$ (Ω)	10–150	95.2
$L_{s}$ (H)$\;\times \;10^{-10}$	1–50	25.7
$C_{s}$ (F)$\;\times \;10^{-15}$	1–50	14.2
$R_{p}$ (Ω)	50–500	358.3
$L_{p}$ (H)$\;\times \;10^{-9}$	1–50	15.8
$C_{p}$ (F)$\;\times \;10^{-14}$	1–50	17.9
Mean Square Error in $R_{in}$	${\mathrm{MSE}}_{Rin}$(%)	0.22%
Mean Square Error in $X_{in}$	${\mathrm{MSE}}_{Xin}$(%)	3.09%

**Table 3:** Performance comparison of the proposed CP-MED antenna with literature.

Ref	$|S_{11}|\;<-10$ dB	Antenna	Antenna	$\lambda_{0}$	AR < 3 dB	Peak	Efficiency
	Range	Size	Size	at $f_{0}$	Range	Gain	(*η*%)
	(GHz)	(mm^3^)	(*λ*_0_)	(GHz)	(GHz)	(dBi)	
[9]	24.1–31 (25%)	10 × 10 × 1.5	0.92×0.92×0.137	27.5	25–30 (18.2%)	8.5	88
[10]	22.6–31.3 (32.2%)	13.2 × 14.8 × 1.524	1.19×1.33×0.137	27	22.6–25.7 (11.5%)	7.8	82
[26]	27.6–31.1 (11.8%)	16 × 9 × 3.016	1.56×0.88×0.296	29.5	28.3–29.7 (4.7%)	7.2	N/A
[24]	19.5–29.8 (42%)	10 × 10 × 1.5	0.82×0.82×0.122	24.5	24.9–28.6 (15.1%)	7.8	N/A
[25]	24.4–38.5 (44.7%)	8 × 8 × 1.747	0.84×0.84×0.183	31.5	26.6–28.9 (7.3%)	6.9	N/A
**Proposed**	**22.97–28.12 (20.2%)**	**15 × 15 × 1.5**	**1.28 × 1.28 × 0.128**	**25.5**	**22.23–27.83 (21.9%)**	**8.8**	**86–93**

**Table 4:** Comparison between the proposed CP MIMO system parameters and other reported works.

Ref	MIMO	Antenna Size	Diversity	$|S_{11}|\;<-10$ dB	$|S_{21}|$	Gain	Efficiency	ECC	DG	MEGC
	Size	(mm^3^)	Type	(GHz)	(dB)	(dBi)	(*η*%)			(dB)
[28]	4	23 × 18 × 0.25	LP	26–40	<−18	4.7–6.6	90	<0.001	9.95	−3.16
		2.53 × 1.98 × 0.027 ($\lambda_{0}^{3}$)								
[29]	2	10 × 14 × 0.8	LP	29.04–31.57	<−20	6.2	98	<0.05	9.99	NA
		1.01 × 1.42 × 0.081 ($\lambda_{0}^{3}$)								
[30]	2	11 × 21.7 × 0.254	CP	25.5–27.79	<−30	5	90	NA	9.91	NA
		0.98 × 1.93 × 0.023 ($\lambda_{0}^{3}$)								
[31]	2	20.5 × 12 × 0.79	CP	25–31	<−24	8.75	97	NA	10	NA
		1.92 × 1.12 × 0.074 ($\lambda_{0}^{3}$)								
**Prop.**	**2**	**15 × 25.8 × 1.5**	**CP**	**22.8–27.5**	**<−23**	**8.9**	**95**	**<0.003**	**9.99**	**<−3**
		**1.28 × 2.32 × 0.125 (** $\lambda_{0}^{3}$ **)**								
**Prop.**	**4**	**25.8 × 27.87 × 1.5**	**CP**	**22–28**	**<−20**	**8.86**	**93**	**<0.001**	**≈10**	**<−3**
		**2.15 × 2.32 × 0.125 ($\lambda_{0}^{3}$)**								

## Data Availability

The original contributions presented in this study are included in the article. Further inquiries can be directed to the authors.
